# How I do it: proximal control in parkinson’s triangle for a very large paraclinoid aneurysm

**DOI:** 10.1007/s00701-021-04961-6

**Published:** 2021-08-14

**Authors:** Victor Volovici, Ruben Dammers

**Affiliations:** Department of Neurosurgery, Erasmus MC Stroke Center, Erasmus MC University Medical Center Rotterdam, Rotterdam, The Netherlands

**Keywords:** Cerebral aneurysm, Microsurgical clip reconstruction, Clipping, Proximal control, Paraclinoid aneurysms

## Abstract

**Background:**

Paraclinoid aneurysms, especially when they are large, can be quite difficult to treat, both endovascularly and through microsurgical clip reconstruction. There are many possibilities to approach this region surgically, and most hinge on total or partial removal of the anterior clinoid process. Gaining proximal control may be a challenge when space is limited, which is why Parkinson’s triangle may be a viable alternative in some cases.

**Methods:**

We describe in a stepwise fashion the steps used to reconstruct a very large paraclinoid aneurysm. We first attempted to gain proximal control in the carotid cave and later in Parkinson’s triangle because of limited manoeuvrability.

**Conclusion:**

Proximal control in Parkinson’s triangle can be a safe alternative when the post-clinoidal segment of the internal carotid artery (ICA) is short and working space is limited in paraclinoid aneurysm microsurgical clip reconstruction.

**Supplementary Information:**

The online version contains supplementary material available at 10.1007/s00701-021-04961-6.

## Introduction

Paraclinoid aneurysms are difficult lesions to treat, both microsurgically and endovascularly [6, 9]. On the one hand, the proximity of important neurovascular structures (the optic nerve, ophthalmic artery, superior hypophyseal artery, posterior communicating and anterior choroidal arteries and oculomotor nerve) limits manoeuvrability, especially in the presence of a large aneurysm. On the other hand, the presence of the anterior clinoid and of the distal dural ring of the clinoidal carotid artery limits options for adequate proximal control.

One pre-requisite for safe dissection and microsurgical clip reconstruction of these lesions is the anterior clinoidectomy, performed in either an extradural [8] or intradural [2] fashion. This step offer access to the distal dural ring and carotid cave and exposes the anterior bend of the carotid in the cavernous sinus [1].

The triangle was described in a case report published in 1965 by Dwight Parkinson [10]. The true pioneer of cavernous sinus surgery is Vinko Dolenc, who in the early 1980s began treating challenging vascular and oncological lesions in and around the cavernous sinus [4]. Despite the fact that indications for these lesions are declining, these pioneers paved the way for current neurosurgeons to tackle challenging aneurysms with a varied armamentarium.

We present the case of a very large paraclinoid aneurysm and posterior communicating artery (PCom) aneurysm, both treated with microsurgical clip reconstruction (RD). We aim to discuss the nuances of achieving proximal control in this setting, but also the pitfalls and potential complications.

## Relevant surgical anatomy

As outlined above, several anatomical structures need to be taken into account. Pre-operative CTA and digital subtraction angiography (DSA) are essential to evaluate the aneurysm neck and dome, the take-off of the PCom and the anterior choroidal artery as well as the direction of the post-clinoidal segment of the ICA. Pre-operative non-contrast CT reveals eventual pneumatization of the anterior clinoid process or the presence of a middle clinoid, carotid canal or clinoid bar [3]. Dural peeling to expose the lateral wall of the cavernous sinus should be practiced in the cadaver lab multiple times before attempting it in the OR. Bleeding in this area can be successfully controlled with fibrin glue [7].

After the petrous portion of the ICA, under the petrolingual ligament and at the posterior border of V3, the carotid artery enters the cavernous sinus. The first intracavernous part ascends towards the upper clivus and dorsum sellae and is joined shortly after cavernous sinus entry by the abducens nerve, the only truly intracavernous cranial nerve. At the level of the dorsum the carotid bends anteriorly, at the “posterior bend”. From this point on, it runs roughly parallel to the middle fossa and anterior clinoid process and can be accessed via Parkinson’s triangle. Under the clinoid, the ICA turns again in its “anterior bend” and pierces the dura at the level of the distal dural ring, which needs to be incised and divided in order to mobilize the carotid and gain access to the carotid cave [5].

## Description of the technique

### Positioning

The patient is positioned prone, with the head turned 45 degrees to the right side and slightly extended. The head is fixed in a Doro® clamp. A classic pterional curvilinear incision is marked, minimal shaving is performed and the skin is draped in standard fashion.

### Incision, soft tissue dissection and craniotomy

The incision is placed in a curvilinear fashion, from the root of zygoma to just above the superior temporal line. Interfascial dissection is performed. The temporal muscle is incised and freed, both from the frontal process of the zygomatic bone anteriorly, posteriorly, following the incision as well as from the root of zygoma. The muscle is then reflected anteriorly and inferiorly, to facilitate a low temporal exposure for the pre-temporal approach.

Three burr holes are placed, one at the keyhole, one posteriorly below the superior temporal line and on the squamosal suture and one as low as possible above the root of zygoma.

### Anterior clinoidectomy

The rest of the surgery is performed under microscopic magnification. After removal of the bone flap, the lateral greater sphenoid wing is drilled away with a Stryker(R) 4 mm cutting drill until the meningo-orbital band is exposed. A small part of the lateral orbital wall and the wall of the superior orbital fissure are removed. The meningo-orbital band is coagulated and cut with a 15 blade in a layer-by-layer fashion, until the dissection plane between the dura propria and the lateral wall of the cavernous sinus is revealed. The dural peeling is performed until foramen rotundum and V2 are identified. We also identify the fourth nerve trajectory and V1. Bleeding from the cavernous sinus is controlled with fibrin glue injected between V1 and V2.

Using a Rhoton® microdissector, we identify the intraorbital entry point of the optic nerve and can safely open the optic nerve canal using fine bone rongeurs. As a final step, the optic strut is drilled until the clinoid tip can be detached completely and the tip is freed from its surroundings and ligaments using a microdissector.

The dura is opened using a linear incision just above the Sylvian fissure, fanning out towards the roof of the cavernous sinus inferiorly and the optic nerve superiorly. By cutting the dura this way, the triangular part which represents the clinoidal dura can be excised, revealing the distal dural ring on the carotid and the take-off of the ophthalmic artery. The distal dural ring is cut flush to the carotid using fine microscissors and the carotid cave and anterior bend of the carotid are exposed.

### Microsurgical clip reconstruction

The paraclinoid and PCom aneurysms are identified, together with the take-off of the ophthalmic artery, the PCom and anterior choroidal arteries and the oculomotor nerve en route to the roof of the cavernous sinus. The PCom aneurysm is reconstructed with two straight Lazic ® clips. Micro-Doppler is used to check the patency of the vessels before and after clip reconstruction.

**Fig. 1:**
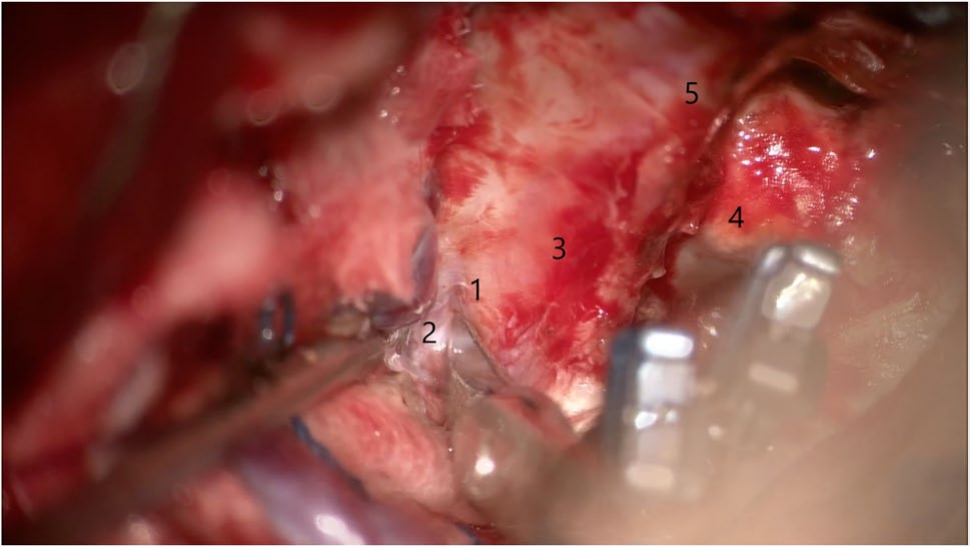
Opening Parkinson’s triangle using sharp dissection. 1 — Trochlear nerve, 2 — Parkinson’s triangle, 3 — oculomotor nerve, 4 — supraclinoid ICA, 5 — distal dural ring

**Fig. 2:**
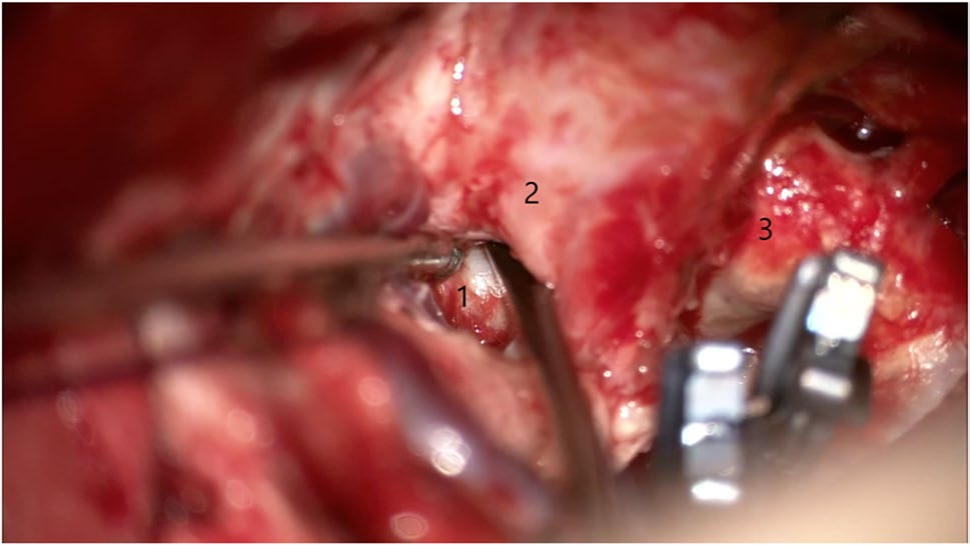
Freeing the horizontal ICA within Parkinson’s triangle. 1 — Horizontal portion of the cavernous ICA, 2 — trochlear nerve, 3 — supraclinoid ICA

**Fig. 3:**
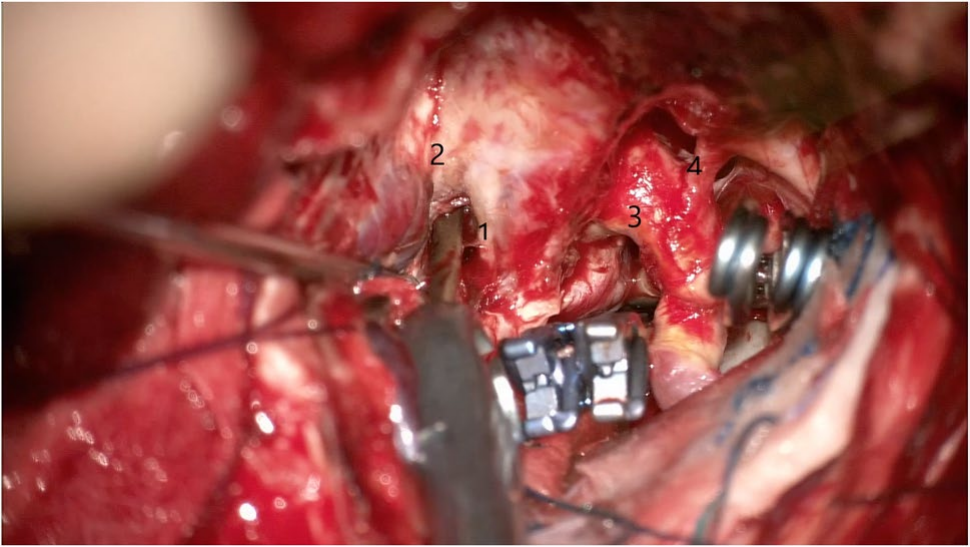
Final position of the clips, removing the temporary clip from Parkinson’s triangle. 1 — Trochlear nerve, 2 — V1, 3 — supraclinoid ICA, 4 — ophthalmic artery

We then attempted to ensure proximal control in the carotid cave. However, due to the aneurysm mass and the short inatracranial post-clinoidal segment of the ICA, placing a proximal clip impeded aneurysm dissection. We decided to incise the lateral wall of the cavernous sinus in its posterior half just underneath the trochlear nerve and above V1(Fig. 1). This opening, which is variable in height, always allows access to the horizontal part of the cavernous ICA (Fig. 2, Operative Video). We could then easily ensure proximal control. We further placed temporary clips on the ophthalmic artery and PCom, A1 and M1 segments, and could reconstruct the paraclinoid aneurysm using large curved Yasargil clips (Fig. 3). Micro-Doppler was used to check the patency of the vessels before and after clip reconstruction.

### Closure

A small piece of abdominal fat was used to cover the dural defect at the level of the clinoid. The dura was closed with 5–0 Prolene. The bone flap was replaced using EvoFix® titanium plates. Muscle and subcutis were closed with 2–0 Vicryl and the skin was closed using an Ethilon® 3–0 running suture.

### Post-operative course

**Fig. 4:**
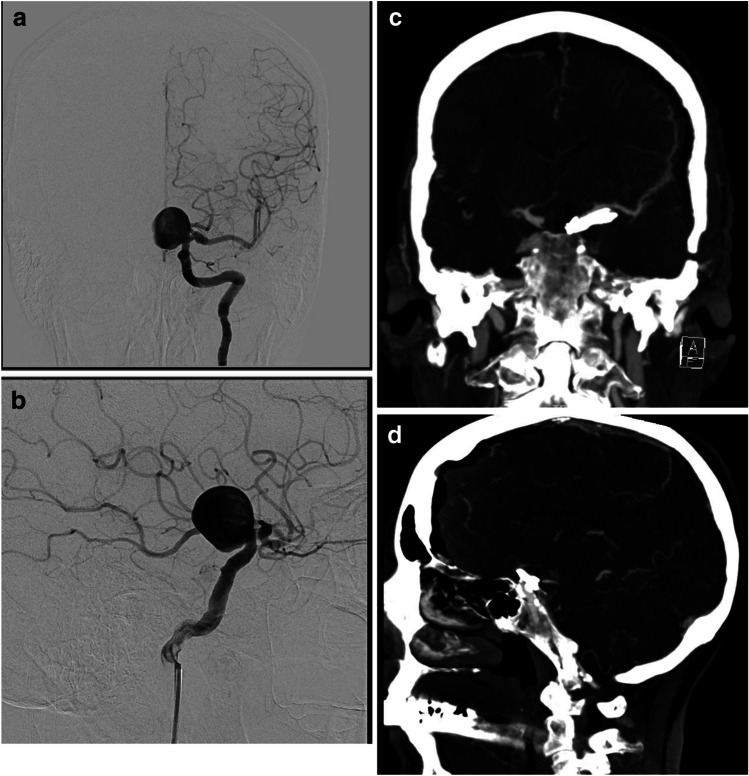
Pre- and post-operative imaging studies.** A** Pre-operative DSA, AP view, revealing a very large paraclinoid aneurysm. **B** Pre-operative DSA, lateral view, showing the very large paraclinoid aneurysm and the PCom aneurysm. **C** Post-operative CTA, coronal view: Complete obliteration of the aneurysm and filling of all blood vessels. **D** Post-operative CTA, sagittal view. Complete obliteration of the aneurysm. Please observe the calcifications inside the carotid at the level of the cavernous sinus and the supraclinoid segment

The post-operative course was uneventful, the patient experienced no cranial nerve deficits or other complications and the post-operative CT angiography revealed patent vessels, no spasm and complete obliteration of the aneurysm (Fig. 4C and D). She was discharged at home on day 3 post-operatively.

## Indications

Aneurysm reconstructions with a very small clinoidal area, where temporary clips impede proper 360 degrees visualization of the aneurysm. Temporary clipping in Parkinson’s triangle can also be used in skull base lesions where iatrogenic ICA injury occurs.

## Limitations

In skull base lesions in which the cavernous sinus is invaded, the horizontal ICA might be difficult to find, especially when it is compressed and displaced medially. An alternative is drilling the floor of the middle skull base fossa and clipping the petrous ICA. In yet other cases, the distance between the trochlear nerve and V1 might be small, which makes dissection more difficult.

Avoiding complications

- Careful pre-operative anatomical evaluation, including CT, CT angiography and DSA
- Evaluating the presence of a middle clinoid, carotid canal or clinoid bar
- Extensive cadaver lab experience in performing all steps described (drilling of the anterior clinoid, peeling the dura of the cavernous sinus)
- Using abdominal fat for closure
- Ensuring proximal control and 360 degrees visualization of the aneurysm after microsurgical clip reconstruction. The aneurysm needs to be cut open at the end to confirm it no longer fills; otherwise, clip reconstruction is not considered successfully confirmed!

Specific perioperative considerations

Pre-operative

- Thin-slice skull base CT, CT angiography, DSA (Fig. 4A and B)
- Hybrid OR if available

Post-operative

- Head elevation 30 degrees
- Systolic blood pressure max 140 mmHg
- Post-operative CT angiography on day 1
- Multidisciplinary discussions in the neurovascular board
- Mobilization without restrictions on day 1

Information for patients: surgery and risks

- Standard pre-operative neurosurgical work-up and patient consent should be performed. Complications should be mentioned, including bleeding (extra- or intradural), infection and CSF fistula
- Given the complexity of the anatomical region, cranial nerve deficits should be discussed, including complete ocular motor palsy
- The risk of infarction in particular should be discussed, either through iatrogenic occlusion or through thromboembolic occlusion, especially in patients with severe atherosclerosis
- All patients should be discussed in the neurovascular board and presented with both microsurgical and endovascular options, if these are available, together with the *previous results of the teams from the particular centre* *they present to.* The discussion should be realistic and focused on the results, and not on unscientific arguments such as “minimally invasive” and “no need to cut the head open”.

## Supplementary Information

Below is the link to the electronic supplementary material.

Supplementary file1 (MPG 107858 KB)

## References

[1] Basma J, Moore KA, Krisht K, Abuelem T, Arnautovic K, Michael LM, Aboud E, Krisht AF (2020). Morphometric comparison of the pterional trans-Sylvian and the pretemporal trans-clinoidal approaches to the posterior communicating artery. Operative neurosurgery (Hagerstown, Md).

[2] Cohen-Gadol A (2021). Intradural clinoidectomy: a tailored osteotomy for approaching the paraclinoid carotid artery and interpeduncular cisterns. World Neurosurg.

[3] Dagtekin A, Avci E, Uzmansel D, Kurtoglu Z, Kara E, Uluc K, Akture E, Baskaya MK (2014). Microsurgical anatomy and variations of the anterior clinoid process. Turk Neurosurg.

[4] Dolenc V (1983). Direct microsurgical repair of intracavernous vascular lesions. J Neurosurg.

[5] Inoue T, Rhoton AL, Theele D, Barry ME (1990). Surgical approaches to the cavernous sinus: a microsurgical study. Neurosurgery.

[6] Kamide T, Misaki K, Uno T, Yoshikawa A, Uchiyama N, Nakada M (2021). Extracranial-intracranial high-flow bypass as a rescue therapy for incomplete cerebral aneurysm occlusion after flow diversion: a case report. Surg Neurol Int.

[7] Krayenbühl N, Hafez A, Hernesniemi JA, Krisht AF (2007) Taming the cavernous sinus: technique of hemostasis using fibrin glue. Neurosurgery 61:E52; discussion E52. 10.1227/01.neu.0000289712.72555.9c 10.1227/01.neu.0000289712.72555.9c 17876221

[8] Krisht AF (2005). Transcavernous approach to diseases of the anterior upper third of the posterior fossa. Neurosurg Focus.

[9] Krisht AF, Hsu SPC (2008) Paraclinoid aneurysms: Part 1: Superior (true ophthalmic) aneurysms. 30:1–5. 10.1097/01.Cne.0000326107.26274.63

[10] Parkinson D (1965). A surgical approach to the cavernous portion of the carotid artery. Anatomical studies and case report. J Neurosurg.

